# Vascular remodeling in arteriovenous fistula treated with PDE5A inhibitors

**DOI:** 10.14814/phy2.70331

**Published:** 2025-04-29

**Authors:** Maheshika Somarathna, Hannah Northrup, Kevin Ingle, Tatyana Isayeva‐Waldrop, Nguyen Thuy Nhu Nguyen, Bailey Lose, Yan‐Ting Shiu, Timmy Lee

**Affiliations:** ^1^ Department of Medicine and Division of Nephrology University of Alabama at Birmingham Birmingham Alabama USA; ^2^ Department of Internal Medicine and Division of Nephrology and Hypertension University of Utah Salt Lake Utah USA; ^3^ Veterans Affairs Medical Center Salt Lake City Utah USA; ^4^ Nora Eccles Harrison Cardiovascular Research and Training Institute, University of Utah Salt Lake City Utah USA; ^5^ Veterans Affairs Medical Center Birmingham Alabama USA

**Keywords:** arteriovenous fistula, hemodialysis, PDE5A, sildenafil, vascular access, vascular remodeling

## Abstract

The arteriovenous fistula (AVF) is the lifeline for hemodialysis patients. However, there are currently no effective therapies promoting AVF maturation. AVF dilation by smooth muscle cell relaxation, through increased cyclic guanosine monophosphate (cGMP), is one potential mechanism to improve AVF remodeling. In this study, we examined the cGMP pathway and its inhibitor phosphodiesterase 5A (PDE5A) in rat, pig, and human AVF. We administered the PDE5A inhibitor, sildenafil, to rats with femoral AVFs and analyzed AVF histological and hemodynamic parameters. We observed that AVF creation increases PDE5A expression in rodent and porcine AVF models. Similarly, we observed an increase in PDE5A expression in the anastomotic regions of AVFs from hemodialysis patients when compared to pre‐AVF placement. Sildenafil‐treated rats showed significantly increased ultrasound‐derived AVF volumetric blood flow and increased MRI‐derived 3‐dimensional lumen diameter when compared to controls. MRI‐based computational fluid dynamics showed that sildenafil‐treated rats had increased anastomotic hemodynamics compared to control rats. Histology showed similar intimal hyperplasia in sildenafil‐treated and control rats. In conclusion, sildenafil treatment increases AVF vein outward expansion and blood flow without affecting the level of intimal hyperplasia. PDE5A inhibitors serve as a potential therapeutic approach to promote AVF maturation by enhancing outward vascular remodeling.

## 1 INTRODUCTION

70% of the over 800,000 patients with end stage kidney disease (ESKD) in the United States use hemodialysis as their main kidney replacement therapy (United States Renal Data System, 2022). Hemodialysis requires a functional and durable vascular access. Arteriovenous fistulas (AVF), the recommended vascular access type (Gold & Hoffman, 2006; Sands, 2000), have high maturation failure rates of up to 60% (Dember et al., 2008).

A successfully matured AVF is defined as one that has an adequate increase in diameter and flow rate, as required for the dialysis, as well as superficial enough to allow for easy cannulation (Lok et al., 2020). Successful AVF remodeling is largely attributed to limited intimal hyperplasia growth and successful outward remodeling. Conversely, excessive intimal hyperplasia development and/or inadequate outward remodeling may limit flow through the AVF, leading to venous stenosis and AVF failure (Lee, 2013; Martinez et al., 2018; Rothuizen et al., 2013; Roy‐Chaudhury et al., 2007; Shiu et al., 2019; Vazquez‐Padron et al., 2021).

The creation of the AVF by the direct anastomosis of the artery and vein redirects arterial blood through the vein, which should result in the needed increase in blood flow rate and outward remodeling. However, AVF creation also produces flow disturbances which may be detrimental to remodeling and lead to insufficient flow increase (Asif et al., 2006; Corpataux et al., 2002; Krishnamoorthy et al., 2008; Rajabi‐Jagahrgh et al., 2013). Smooth muscle cell (SMC) relaxation may play a role in the AVF remodeling process by accommodating these hemodynamic changes (Pike et al., 2019; Shiu et al., 2019; Somarathna et al., 2022), specifically through the increase of cyclic guanosine monophosphate (cGMP). cGMP leads to SMC relaxation primarily through protein kinase G (PKG) activation and the subsequent downstream events that result in decreasing intracellular calcium levels (Birschmann & Walter, 2004; Denninger & Marletta, 1999; Montfort et al., 2017). PDE5A impairs this process by degrading cGMP (Corbin, 2004; Lin, 2004; Omori & Kotera, 2007). Specifically, PDE5A degrades cGMP by catalyzing the hydrolysis of a phosphodiester bond in cGMP, which converts it to its inactive form, 5′‐guanosine monophosphate (Ahmed et al., 2021; Lugnier, 2006).

In the present study, we first investigated the protein expression of PDE5A following AVF creation in a rodent model, a porcine model, and hemodialysis patients. Next, we treated AVF with a PDE5A inhibitor, sildenafil, prior to and after AVF creation in a rodent model. We hypothesized that pre‐treatment with sildenafil prior to AVF creation and after may be a therapeutic approach to promote successful AVF development.

## 2 METHODS

### 2.1 Rat studies

All experiments were performed in accordance with the National Institutes of Health guidelines, the US National Research Council's “Guide for the Care and Use of Laboratory Animals,” the US Public Health Service's “Policy on Humane Care and Use of Laboratory Animals” and “Guide for the Care and Use of Laboratory Animals”, and approved by the University of Alabama at Birmingham Institutional Animal Care and Use Committee. Sildenafil citrate (Fisher Scientific CAS# 171599–83‐0) was dissolved in a solution of sucrose (5.7 mg/kg) and 100% ethanol (2.8 μL/mL) to reach a final dose of 90 mg per rat. It was administered through daily drinking water to male, 12–16 week‐old Sprague Dawley rats (Taconic Biosciences, Hudson, NY) for 14 days. Controls received a similar solution of sucrose (5.7 mg/kg) and 100% ethanol (2.8 μL/mL) which was administered through daily drinking water. The methods and drug concentration were chosen based on Sasser et al. which found that 90 mg/kg of sildenafil treatment in rats resulted in the largest increase in cGMP concentrations while still remaining safe for the animals (Sasser & Baylis, 2010). All animals were fed a standard rodent lab chow (LABDIET NIH‐31 TAC AUTO/IRRADIATED) provided by the University of Alabama at Birmingham Animal Resources Program. Femoral side (artery) to end (vein) AVF were then created as previously published (Somarathna et al., 2022). Anesthetics were carprofen, isoflurane, and buprenorphine. Non‐AVF controls consisted of the contralateral femoral vessels. Sildenafil treatment was continued following AVF creation surgery for 7 or 28 days at which time rats were sacrificed for analysis. Rats were euthanized by exsanguination under anesthesia. Briefly, rats were anesthetized under 2% isoflurane in a 100% oxygen mix. The inferior vena cava was then perfused with either PBS (for protein studies) or 10% formalin (for histology). The vessels were exsanguinated as the primary euthanasia method. A cervical dislocation was performed as a secondary euthanasia method after perfusion. AVF vein sections were obtained from 0 to 6 mm proximal to the anastomosis.

### 2.2 Pig studies

All experiments were performed in accordance with the National Institutes of Health guidelines, the US National Research Council's “Guide for the Care and Use of Laboratory Animals,” the US Public Health Service's “Policy on Humane Care and Use of Laboratory Animals” and “Guide for the Care and Use of Laboratory Animals”, and approved by the University of Alabama at Birmingham Institutional Animal Care and Use Committee. 50 kg male Yorkshire cross pigs (Snyder Farms, Southside, AL) underwent AVF creation surgery of the right carotid artery (side) to jugular vein (end). Animals were monitored and then sacrificed for tissue collection 7 days after AVF creation. Pigs were anesthetized under similar conditions as rats but with atropine, telazol, xylazine, and isoflurane. Pentobarbital was injected intravenously at 120 mg/kg and death was confirmed by ECG. Next, vessels were removed for analysis.

### 2.3 Human specimens

Collection of human tissues was approved by the University of Alabama at Birmingham Institutional Review Board and written informed consent was obtained from all participants. Vein segments were collected from patients undergoing two‐stage AVF creation using standard operating procedure. Vein sections that are normally discarded during standard AVF creation were collected during the first stage of AVF creation surgery (pre‐AVF) as well as from the AVF anastomosis region during the second stage transposition surgery (post‐AVF). Upon collection, tissue sections were fixed in formaldehyde to be used for histological staining.

### 2.4 Morphometric analysis

Morphometric analysis of AVF veins from rats was performed as described previously (Somarathna et al., 2022). Briefly, following tissue processing, the AVF vein was divided into three 5 mm segments. From each vein segment, 10–12 slides (each with 5 μm thickness) were obtained by selecting the first of every 10 tissue sections, followed by Verhoeff Van Gieson (Somarathna et al., 2022) and Russell‐Movat pentachrome (Lee et al., 2014) staining. Morphometric analysis was performed using Cellsense Dimension Software (Olympus Life Science) to measure the intimal and medial areas as previously published (Somarathna et al., 2022). Results were reported as the ratio of intimal area to medial area.

### 2.5 Ultrasound analysis

Ultrasound of AVF and contralateral vessels in rats was performed using the VEVO 2100 ultrasound system with a 24–30 MHz bifrequency transducer (Visual Sonics, Toronto, Canada) with B mode color Doppler used to locate the vessels and M mode to measure vessel diameters. Vessel diameter was categorized as (1) 1–3 mm, (2) 3–6 mm, and (3) 6–11 mm away from the anastomosis.

Volumetric blood flow was calculated as follows:

(1) $$\begin{array}{c} \text{Stroke volume}\;\left(\mathrm{\mu L}\right)=\text{velocity time integral}\;\left(\mathrm{mm}\right)\\ \;\times \;0.75\times \text{vessel diameter}{\left(\mathrm{mm}\right)}^{2} \end{array}$$

(2) $$\begin{array}{c} \text{Vessel volumetric flow}\;\left(\frac{\mathrm{mL}}{\min}\right)=\text{stroke volume}\;\left(\mathrm{\mu L}\right)\\ \;\times \;\text{heart rate}\;\frac{\mathrm{bpm}}{1000} \end{array}$$

### 2.6 Immunofluorescence localization of PDE5A

Immunofluorescence staining was performed for PDE5A (Abcam, Cambridge MA, #Ab64179) and smooth muscle actin (DAKO, #M0851) with antigen retrieval using a citrate buffer (pH 6), microwaved for 15 min. Secondary antibodies were goat anti‐rabbit IgG (H + L) cross‐adsorbed secondary antibody, Alexa Fluor™ 594 (Thermo Scientific, cat # A‐11012) and goat anti‐mouse IgG (H + L) cross‐adsorbed secondary antibody, Alexa Fluor™ 488 (Thermo Scientific, cat # A‐11001).

### 2.7 CFD simulation and post‐processing

Rats (4 control and 4 sildenafil treated) were subject to non‐contrast magnetic resonance imaging (MRI) 21 days after AVF creation surgery using a 9.4 Tesla Bruker BioSpec 94/20 MRI Machine (Bruker Biospin, Billerica, MA). CFD simulations were performed, and results were reported as described in our previous studies (Northrup et al., 2021; Somarathna et al., 2022) with some modification for data extraction and analysis. Black‐blood MR images were segmented in Amira (Thermo Fisher Scientific, Waltham, Mass) to reconstruct the lumen into a 3D STL file. The surface was smoothed at a lambda of 0.6 with 60 iterations. Flow extensions were added to the geometry to prevent entrance effects at the region of interest using the Vascular Modeling Toolkit (VMKT) (available at: www.vmtk.org). Centerlines of the fistula vein were calculated in VMTK with points at 0.1 mm intervals starting at the venous segment of the anastomosis and ending 10 mm away from the anastomosis into the fistula vein. MATLAB (MATLAB, Natick, Mass) was used with the centerlines and STL to calculate the diameter of the vein at 0.1 mm increments.

The geometry was meshed in ANSYS ICEM CFD 2019 R3 (Asys, Inc., Canonsburg, PA) with 4 prism layers at the wall and 1.5 × 10^6^ tetrahedra in the lumen domain. Mesh independence studies were previously performed and published (Pike et al., 2017). The velocity was extracted using the 2D gradient echo velocity mapping scans with ImageJ (https://imagej.net/ij/). Velocity scans were located at the proximal artery and fistula vein.

CFD simulations were run using ANSYS Fluent 2019 R3 (Ansys, Inc., Canonsburg, PA). Simulations were transient, with a rigid vessel wall, no‐slip conditions at the wall, and laminar, Newtonian, and incompressible blood flow. Velocity boundary conditions were prescribed at the fistula vein and artery proximal to the heart using the extractions described above. A pressure outlet with a gauge pressure of 0 was set at the distal artery. Blood was prescribed with a viscosity of 0.0035 Pa·s and a density of 1050 kg/m^3^. The SIMPLE scheme had spatial discretization gradient set to Least Squares Based, Pressure set to Second Order, and Momentum set to Second Order Upwind. The transient formulation was second order implicit. Simulations were run for 3 cardiac cycles, with a time step equal to 1/10th of the velocity boundary condition. Data was extracted from the last cardiac cycle.

Data was analyzed using Tecplot 360 (Tecplot, Bellevue, Wa). A slice was created at the anastomosis region, and data (fluid‐wall shear stress (WSS) and vorticity) from the slice were extracted and presented throughout the cardiac cycle.

### 2.8 Western blotting analysis

Western blotting was performed on AVF veins and contralateral vessels of rats. Tissues were crushed in liquid nitrogen and cold radioimmunoprecipitation assay lysis buffer (Millipore, RIPA Lysis Buffer, cat# 20–188) with the addition of protease and phosphatase inhibitors (Thermo Scientific, Halt™ Protease and Phosphatase Inhibitor Cocktail, cat# 78440) used for cell lysis. A 4%–15% polyacrylamide gel (Bio‐Rad, Hercules, CA) was used to separate proteins, followed by transfer to a nitrocellulose membrane (Thermo Scientific, Waltham, MA). Blocking was performed by incubating the membranes with a 5% BSA solution for 1 h, followed by incubation with primary antibodies for PDE5A (BIOSS, cat# BS‐2349R). Secondary antibodies were goat anti‐rabbit IgG (H + L), HRP (Thermo Scientific, cat# 31460). Enhanced chemiluminescent substrate (Thermo Scientific, Waltham, MA) was used to detect protein bands. To quantify total protein expression, densitometric analysis was completed using Gene tools software, with bands normalized to glyceraldehyde‐3‐phosphate dehydrogenase (Cell Signaling, GAPDH (14C10) rabbit mAB, cat# 3693). The full‐length western blots can be seen in Figures S1 and S2.

### 2.9 Statistical analysis

A paired *t*‐test (if data followed a normal distribution), Wilcoxon signed‐rank test (if data did not follow a normal distribution), or a 2‐way ANOVA and Tukey's multiple comparisons test was used for group analysis, with statistical significance set to *p < 0.05 and **p < 0.01. All data are presented as mean ± S.D.

## 3 RESULTS

**FIGURE 1:**
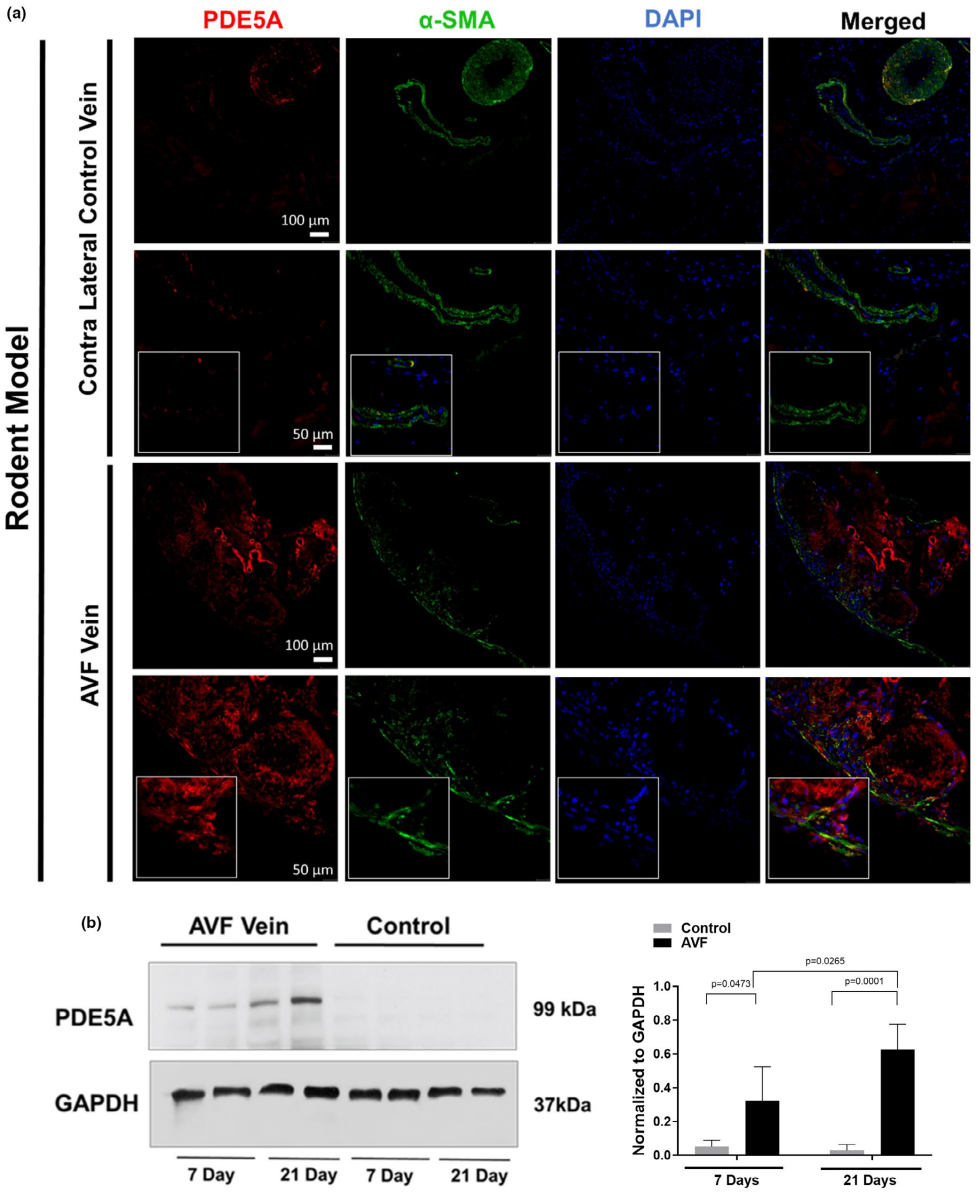
PDE5A expression after AVF creation surgery in rats. (a) Representative immunofluorescence of PDE5A, smooth muscle actin (α‐SMA), and DAPI in contralateral control veins and AVF veins 7 days after AVF creation surgery. Boxed images are at 40x magnification. (b) Representative western blot and densitometric analysis (*n* = 4/group).

**FIGURE 2:**
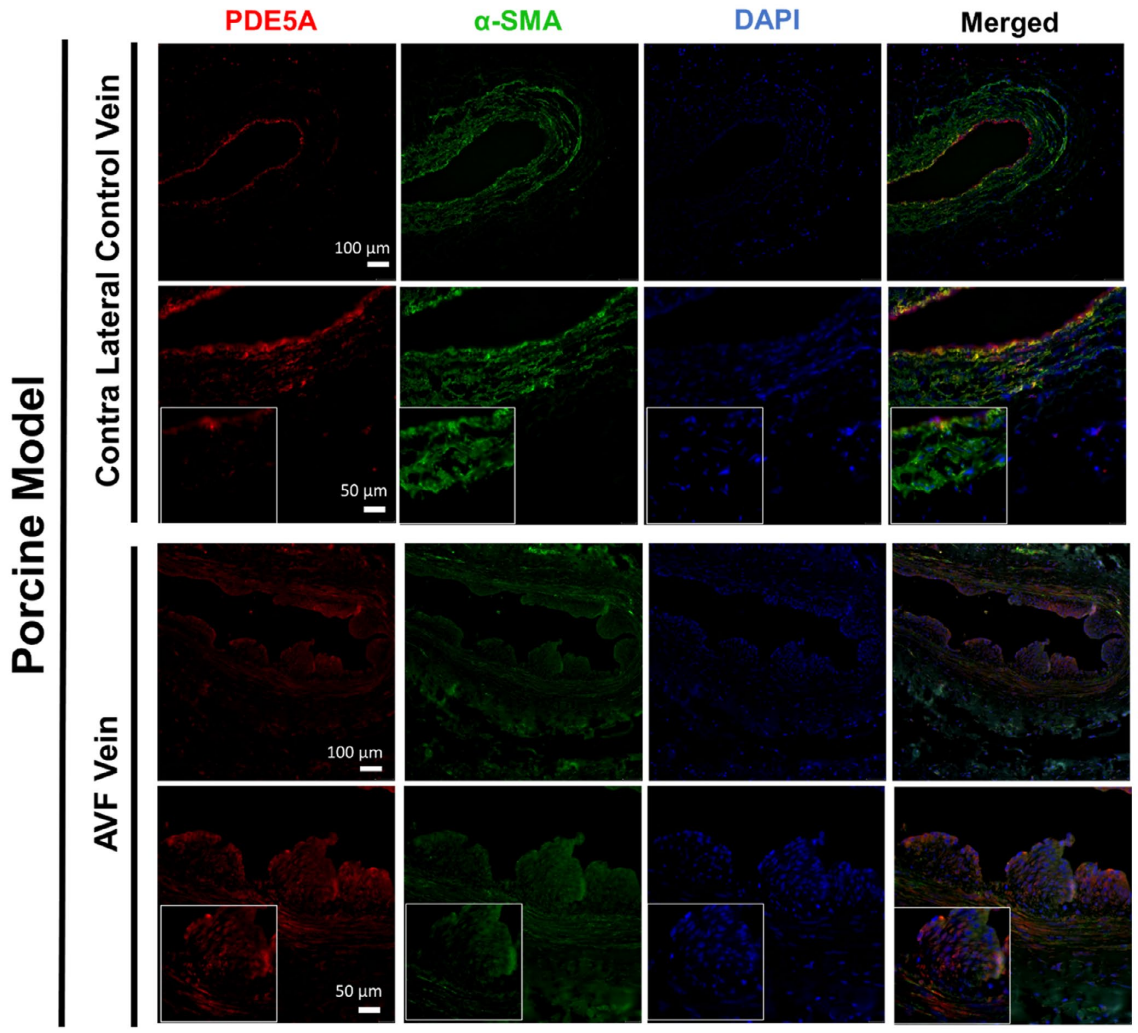
PDE5A expression after AVF creation surgery in pigs. Representative immunofluorescence staining of PDE5A, smooth muscle actin (α‐SMA), and DAPI in contralateral control veins and AVF veins 7 days after AVF surgery. Boxed images are at 40x magnification.

**FIGURE 3:**
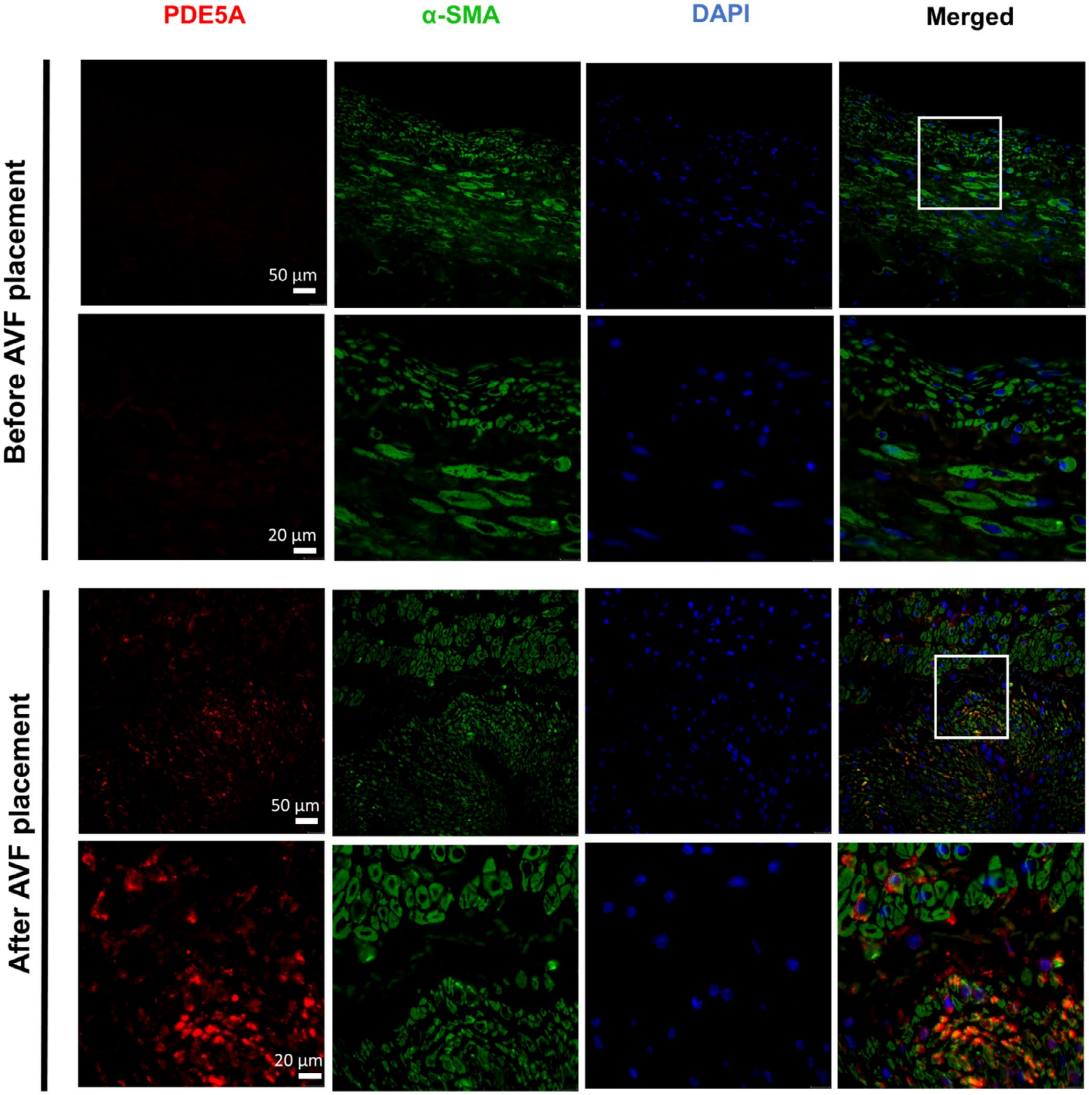
PDE5A expression in ESKD patient tissues before and after AVF surgery. Representative immunofluorescence staining of PDE5A, smooth muscle actin (α‐SMA), and DAPI from the vein prior to AVF creation surgery (1st stage) and at the AVF anastomotic region after AVF creation surgery (2nd stage).

**FIGURE 4:**
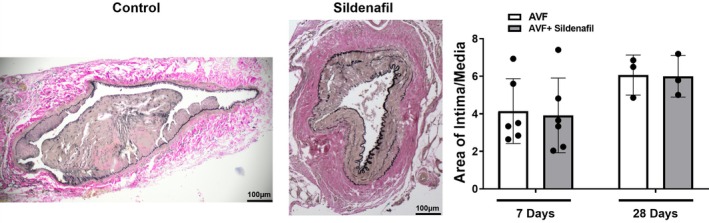
Intimal hyperplasia in rats after sildenafil treatment. Left panels show representative images of Russell‐Movat pentachrome staining of control (left) and sildenafil (middle) treated rats. Right panel shows intimal hyperplasia quantification as the ratio of intima to media area from AVF veins of control and sildenafil treated rats 7 and 28 days after AVF surgery (*N* = 3‐6/group).

As demonstrated by western blot, rat PDE5A levels at 7 and 21 days after AVF creation were increased in AVF versus control veins (*p* < 0.05) (Figure 1). Expression of PDE5A by immunostaining was also confirmed in the intima and media layers of both rat and pig vessels at 7 days after AVF creation but was limited in contralateral vessels (Figures 1 and 2). In patients, PDE5A expression was observed to be higher in the AVF anastomosis from the second stage of AVF creation compared to the first stage (pre‐AVF placement) (Figure 3). However, morphometric analysis did not reveal any significant differences in the venous anastomosis average intima/media ratio comparing sildenafil to control treated rats, which suggests that intimal hyperplasia is not affected by PDE5A inhibition (Figure 4).

**FIGURE 5:**
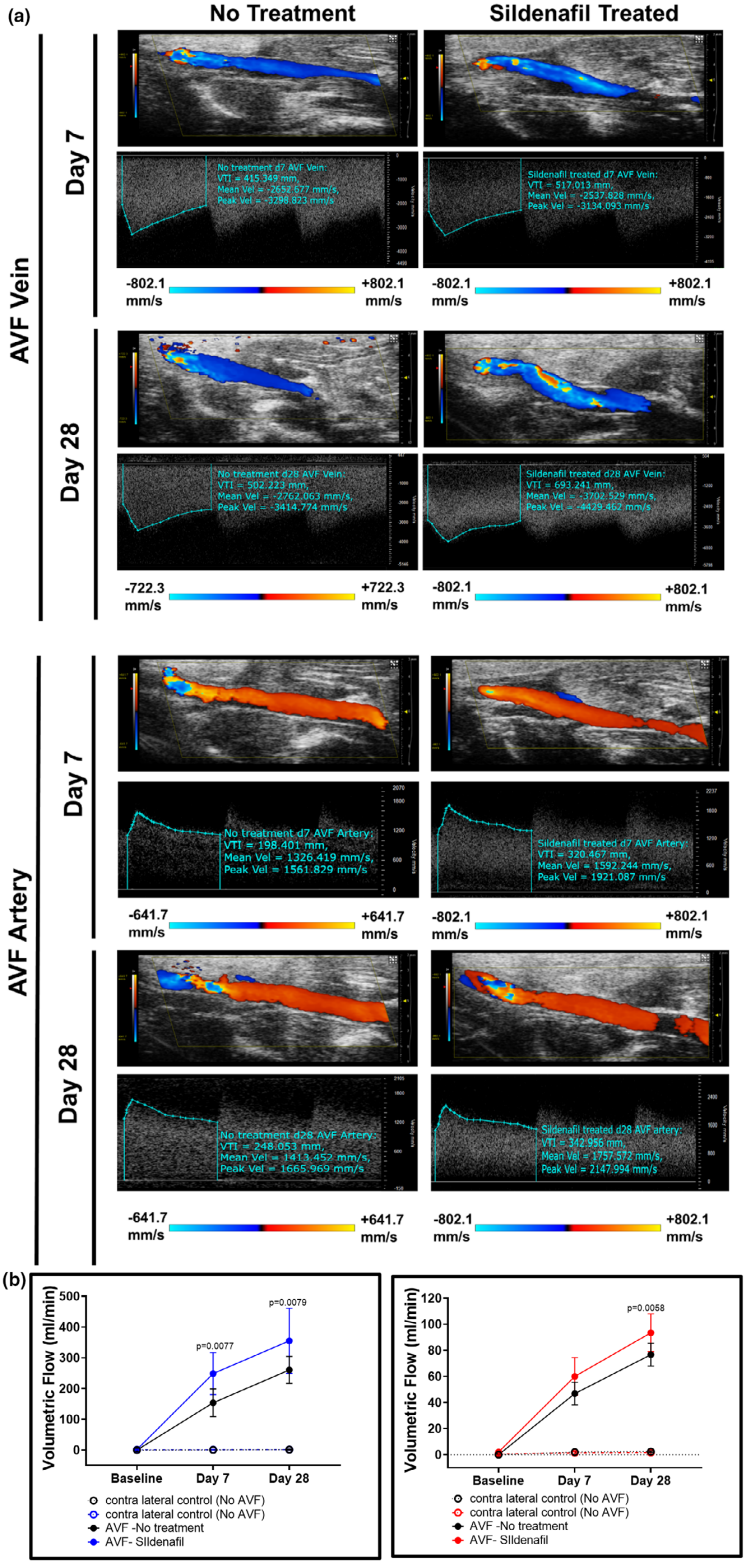
Ultrasound and volumetric flow of sildenafil‐treated rats. (a) Representative B mode Doppler color images and waveform of AVF vein and arteries from sildenafil and control treated rats. (b) Volumetric blood flow (mL/min) quantification of vein (left panel) and artery (right panel). *N* = 6 in each group. Exact *p* value is for AVF – No treatment vs. AVF – Sildenafil. *p* < 0.0001 for AVF – No treatment and AVF – Sildenafil compared to contralateral controls at Day 7 or Day 28. In this figure, “No treatment” refers to control‐treated rats.

**FIGURE 6:**
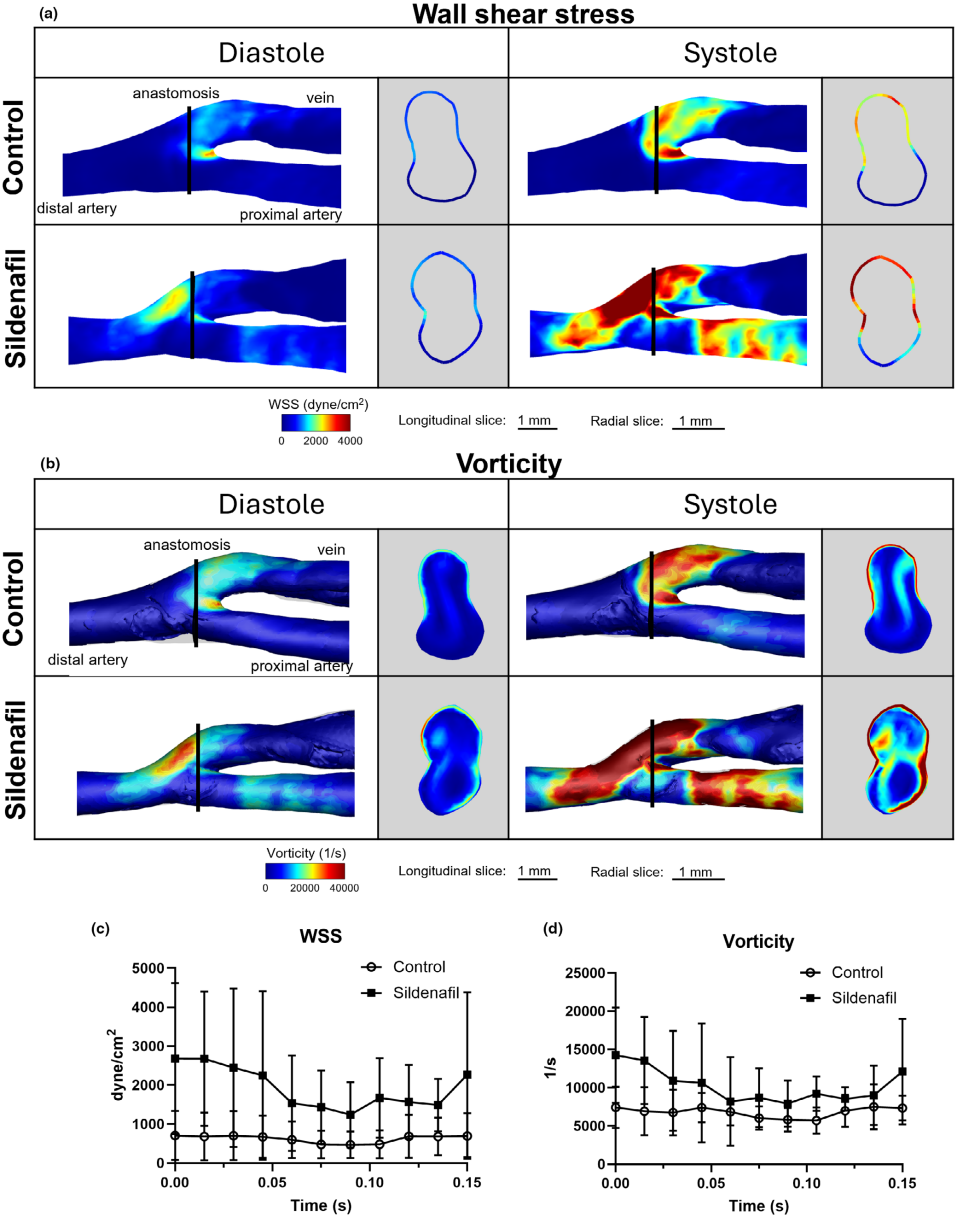
Hemodynamics of rat AVF at 21 days after creation surgery. (a) Wall shear stress (WSS) and (b) vorticity color maps of longitudinal and radial slices at systole and diastole. The black line in the longitudinal slices show location of respective radial slices. (c) WSS and (d) vorticity over a cardiac cycle. Data in (c, d) extracted from radial slices in (a, b). Error bars show ± SD. Control was significantly different from sildenafil when averaged over the cardiac cycle (*p* < 0.001, *n* = 44 data points; 4 rats/group).

Ultrasound of rat AVF at 7 and 28 days following AVF creation showed a significant increase in volumetric blood flow in both the inflow artery and the outflow vein of the AVF following sildenafil treatment when compared to control (*p* < 0.05) (Figure 5). Similarly, MRI‐derived 3‐dimensional diameter showed an increase with sildenafil treatment (1.75 ± 0.45 mm) compared to control‐treated rats (1.54 ± 0.29 mm) (*p* < 0.001). CFD results also showed that sildenafil‐treated rats had higher wall shear stress and vorticity throughout the cardiac cycle in AVFs compared to control rats (*p* < 0.001) (Figure 6).

## 4 DISCUSSION

This study explored the role of PDE5A in AVF, showing that PDE5A expression is increased in AVF of rats, pigs, and patients when compared to native veins. We then investigated PDE5A as a potential target for improved AVF remodeling through the use of the PDE5A inhibitor, sildenafil, administered orally to rats with femoral AVFs. We demonstrated that rats with sildenafil treatment showed increased volumetric blood flow and hemodynamics. This research contributes to the current knowledge of AVF remodeling pathways. Specifically, cGMP and its inhibitor PDE5A offer a potential therapy for improved AVF remodeling through promoting outward remodeling.

AVF maturation is dependent on a combination of outward lumen expansion and limited inward remodeling or intimal hyperplasia growth. The absence of approved therapies outside of intravascular interventions signifies an insufficient understanding of the mechanisms behind AVF remodeling. We have previously shown the importance of the cGMP pathway through nitric oxide synthase knockout and overexpression mouse AVF models as well as the application of a nitric oxide releasing gel to rats with femoral AVF. Those studies showed that nitric oxide has a limiting effect on intimal hyperplasia while increasing outward remodeling (Somarathna et al., 2022). Nitric oxide is an upstream regulator in the cGMP pathway, that is, nitric oxide increases cGMP, and in the present study we decided to look more downstream in the pathway, at the cGMP inhibitor PDE5A. PDE5A is an ideal therapeutic target as inhibitors, such as sildenafil, act as a vasodilator and have long been approved for other indications.

Our ultrasound results show an increase in volumetric blood flow rate in AVF compared to contralateral controls. Such an increase, when compared to non‐surgery controls, may be due to several factors, including increased cardiac output, decreased resistance in the AV shunt due to bypassing the downstream circulation, and retrograde flow in the distal artery. Indeed, the creation of fistula increases cardiac output in humans by 10%–30% (Dundon et al., 2014; Ori et al., 2002; Rao et al., 2019). Retrograde flow in the distal artery also occurs in patients (Alsaadi, 2022; Sivanesan et al., 1998). Langer et al. used ultrasound to measure rat femoral AVF velocity and reported peak systolic velocity at the anastomosis to be in the range of 1–9 m/s (Langer et al., 2010). This is of the same order of magnitude as our data, with mean and peak venous velocity ranging from about 2.5–3.7 m/s and 3.1–4.4 m/s, respectively. Additionally, our study analyzed hemodynamic parameters at several time points throughout the cardiac cycle, instead of the more common time‐averaged method. This allows us to see how both the vorticity and WSS change over the cardiac cycle. Our sildenafil‐treated rats showed a larger range in values over a cardiac cycle than the controls. This may have been due to the effects of AVF creation as previously discussed or overall systemic effects of PDE5A inhibition.

Our results show that PDE5A is increased after AVF creation. PDE5A is activated by cGMP binding. Additionally, the PDE5A promotor is stimulated by cGMP and cAMP (Omori & Kotera, 2007). However, disturbed flow, such as that seen in the anastomosis of the AVF, is known to decrease nitric oxide, which would ultimately decrease cGMP. It is possible that the observation of increased PDE5A in AVF veins, when compared to native veins, could be a result of a change in blood flow or increased inflammation after surgery (Massaro et al., 2014). Thus, the exact mechanism of how blood flow may impact the expression or activation of PDE5A is unclear and should be studied further.

Previous studies have found that tissue injury associated with oxidative stress and endothelial dysfunction can be reduced by intracellular cGMP (Abdollahi et al., 2003; Dias et al., 2005). Additionally, Veres et al. showed that vein‐grafts preconditioned with PDE5A inhibitors reduce oxidative stress and enhance endothelial function, giving a protective effect from ischemia reperfusion and surgical injury (Veres et al., 2018). Based on these studies, we preconditioned the rats with sildenafil treatment 2 weeks prior to AVF creation surgery and continued treatment until sacrifice. While our study does show improved remodeling, additional studies are needed to understand the pathology and protective effects of PDE5A inhibition on AVF remodeling. Studies are also needed to analyze the dose‐dependent response of AVF remodeling to sildenafil treatment. Additionally, because PDE5A inhibition prevents the degradation of cGMP, mechanistic studies could be done to investigate the impact of PDE5A inhibition on cGMP expression in the context of AVF remodeling.

Previously we found that sildenafil treatment generally increased AVF hemodynamics, with the highest value being near the anastomosis; however, we did not analyze the anastomosis itself (Northrup et al., 2021). In this study, our MRI‐based CFD studies were focused on the anastomosis because this region is susceptible to stenosis (Mortamais et al., 2013). We found that the WSS and vorticity were significantly higher in the sildenafil treatment group when compared to the control at the anastomosis and throughout the cardiac cycle. This is similar to the Hemodialysis Fistula Maturation Consortium ancillary study showing that higher levels of WSS shortly following AVF creation were associated with increased lumen area (He et al., 2023). We also saw a similar trend with WSS in our clinical study comparing patients requiring intervention prior to successful dialysis with those not requiring intervention (i.e., WSS was higher in AVFs not requiring intervention than WSS in AVFs requiring intervention at 1 day after AVF creation surgery) (Northrup et al., 2022). Thus, high WSS immediately following AVF creation may be beneficial for remodeling. In both clinical studies (He et al., 2023; Northrup et al., 2022), WSS decreased with time. The current animal CFD study only had one time point. Additional time points are required to reveal whether WSS in rats and with sildenafil treatment decreases with time.

In this study, vorticity was higher in our sildenafil treated rats. Vorticity is an indicator of disturbed flow by quantifying fluid rotation and has been associated with inflammation and vascular stiffness (Schäfer et al., 2017). In the aforementioned clinical study, as with WSS, vorticity was higher in AVFs not requiring intervention than vorticity in AVFs requiring intervention at 1 day after AVF creation surgery (Northrup et al., 2022). It is possible that the beneficial effect of high WSS on AVF remodeling is a stronger factor than potential negative effects from high vorticity. Future research is necessary to analyze the individual roles of WSS and vorticity in AVF remodeling.

Our results show no significant difference in intimal hyperplasia with sildenafil treatment compared to controls. However, in an arterial injury model, Yang et al. found that PDE5A inhibition reduced hyperplasia compared to controls (Yang et al., 2019). Hirshberg et al. showed similar results to Yang et al. in a carotid endarterectomy model (Hirschberg et al., 2009). The pathologies of veins and arteries differ and may explain the difference in results. Additional studies are needed to understand the role of PDE5A in intimal hyperplasia in the setting of venous versus arterial injury. Finally, time points beyond 28 days are needed to investigate whether neointimal hyperplasia develops in the sildenafil treatment group after a longer duration following AVF creation surgery.

While this study is focused on the PDE5A/cGMP pathway, other mechanisms may also be involved in the remodeling process. Brahmbhatt et al. reviewed several possible mechanisms such as inflammation, uremia from chronic kidney disease (CKD), hypoxia, surgical injury from AVF creation surgery, and thrombosis (Brahmbhatt et al., 2016; Gorecka et al., 2019). Gorecka et al. also cited extracellular matrix remodeling, growth factor secretion, and cell adhesion molecule upregulation as important mechanisms in the AVF remodeling process (Gorecka et al., 2019).

Our present study is limited to data collected 7, 21, and 28 days following AVF creation. Additional study points, both immediately following AVF creation and longer time points, would be beneficial in understanding the relationship between PDE5A inhibition and cGMP with vascular remodeling and hemodynamics. We did not investigate whether sildenafil treatment alone affects PDE5A expression in control AVF vessels. However, Kniotek et al. found that sildenafil downregulated PDE5 expression in human natural killer cells (Kniotek et al., 2021), so it is possible that sildenafil not only inhibits PDE5A activity but also downregulated PDE5A expression. This is an interesting question for future studies. Future studies can also include RNAseq and proteomics to characterize the molecular mechanism associated with the PDE5A therapies and AVF remodeling. Lastly, another limitation is that our animal studies were performed in healthy animals and not in animals induced with CKD, the milieu that is present in hemodialysis patients.

## 5 CONCLUSION

Our study demonstrated that PDE5A is expressed in rat, pig, and patient following AVF creation. Additionally, we showed in a rat AVF model that while PDE5A inhibition therapy does not affect intimal hyperplasia, it does increase blood flow rate, lumen diameter, and hemodynamics. Specifically, our CFD results showed that sildenafil‐treated rats had increased WSS and vorticity over the cardiac cycle in the AVF vein compared to control rats; however, we observed similar intimal hyperplasia in sildenafil‐treated and control rats. Thus, the increase in hemodynamics may be due to the increased AVF volumetric blood flow in sildenafil‐treated rats. Overall, our result indicates that sildenafil treatment increases AVF vein outward expansion. Consequently, PDE5A inhibitors may serve as a potential therapeutic approach to promote AVF maturation by enhancing outward vascular remodeling; however, alternative approaches are needed to limit inward remodeling. This research lays the groundwork for potential therapy in patients to improve AVF maturation.

## AUTHOR CONTRIBUTIONS

Study design: TL, YTS, and MS. Ultrasound: KI. Histology processing and staining: NN, BL, TIW. Western blotting: TIW, BL. CFD procedure and analysis: HN. Drafting manuscript: SM, HN, TL, and YTS. Manuscript review: HN, TL, YTS, NN, BL, TIW.

## FUNDING INFORMATION

This work was supported by National Institutes of Health (NIH) Grants F31DK123977 (Somarathna), R01HL139692 (PI: Timmy Lee), and R01HL153244 (PI: Timmy Lee); and Department of Veterans Affairs Merit Award I01BX003387 and I01BX004133 (PI: Timmy Lee).

## CONFLICT OF INTEREST STATEMENT

Timmy Lee is a consultant for BD Bard, Boston Scientific, Xeltis, and Venostent. All other authors have no competing interests.

## ETHICS APPROVAL

This study was approved by the University of Alabama at Birmingham institutional review board and the University of Alabama at Birmingham institutional animal care and use committee.

## CONSENT TO PARTICIPATE

Written informed consent was provided by all patients.

## DECLARATIONS

The majority of this work has been published by The University of Alabama at Birmingham ProQuest Dissertations Publishing as part of the first author Maheshika Somarathna's dissertation, “The Role of Nitric Oxide and Cyclic Guanosine Monophosphate Signaling in Arteriovenous Fistula Maturation” per Ph.D. graduation requirements.

## Supporting information


Figure S1.

## Data Availability

The datasets used and/or analyzed during the current study are available from the corresponding author on reasonable request.

## References

[phy270331-bib-0001] Abdollahi, M. , Fooladian, F. , Emami, B. , Zafari, K. , & Bahreini‐Moghadam, A. (2003). Protection by sildenafil and theophylline of lead acetate‐induced oxidative stress in rat submandibular gland and saliva. Human & Experimental Toxicology, 22, 587–592. 14686481 10.1191/0960327103ht399oa

[phy270331-bib-0002] Ahmed, W. S. , Geethakumari, A. M. , & Biswas, K. H. (2021). Phosphodiesterase 5 (PDE5): Structure‐function regulation and therapeutic applications of inhibitors. Biomedicine & Pharmacotherapy, 134, 111128. 10.1016/j.biopha.2020.111128 33348311

[phy270331-bib-0003] Alsaadi, M. J. (2022). Arteriovenous fistula anastomosis diameter association with the ischemic steal syndrome occurrence. Open Access Macedonian Journal of Medical Sciences, 10, 2621–2626.

[phy270331-bib-0004] Asif, A. , Roy‐Chaudhury, P. , & Beathard, G. A. (2006). Early arteriovenous fistula failure: A logical proposal for when and how to intervene. Clinical Journal of the American Society of Nephrology: CJASN, 1, 332–339. 10.2215/CJN.00850805 17699225

[phy270331-bib-0005] Birschmann, I. , & Walter, U. (2004). Physiology and pathophysiology of vascular signaling controlled by guanosine 3′,5′‐cyclic monophosphate‐dependent protein kinase. Acta Biochimica Polonica, 51, 397–404. 15218537

[phy270331-bib-0006] Brahmbhatt, A. , Remuzzi, A. , Franzoni, M. , & Misra, S. (2016). The molecular mechanisms of hemodialysis vascular access failure. Kidney International, 89, 303–316. 10.1016/j.kint.2015.12.019 26806833 PMC4734360

[phy270331-bib-0007] Corbin, J. D. (2004). Mechanisms of action of PDE5 inhibition in erectile dysfunction. International Journal of Impotence Research, 16, S4–S7. 15224127 10.1038/sj.ijir.3901205

[phy270331-bib-0008] Corpataux, J. M. , Haesler, E. , Silacci, P. , Ris, H. B. , & Hayoz, D. (2002). Low‐pressure environment and remodelling of the forearm vein in Brescia‐Cimino haemodialysis access. Nephrology, Dialysis, Transplantation, 17, 1057–1062. 10.1093/ndt/17.6.1057 12032197

[phy270331-bib-0009] Dember, L. M. , Beck, G. J. , Allon, M. , Delmez, J. A. , Dixon, B. S. , Greenberg, A. , Himmelfarb, J. , Vazquez, M. A. , Gassman, J. J. , Greene, T. , Radeva, M. K. , Braden, G. L. , Ikizler, T. A. , Rocco, M. V. , Davidson, I. J. , Kaufman, J. S. , Meyers, C. M. , Kusek, J. W. , Feldman, H. I. , & Dialysis Access Consortium Study Group . (2008). Effect of clopidogrel on early failure of arteriovenous fistulas for hemodialysis. A randomized controlled trial. JAMA, 299(18), 2164–2171. 10.1001/jama.299.18.2164 18477783 PMC4943222

[phy270331-bib-0010] Denninger, J. W. , & Marletta, M. A. (1999). Guanylate cyclase and the ⋅NO/cGMP signaling pathway. Biochimica et Biophysica Acta ‐ Bioenergetics, 1411(2‐3), 334–350. 10.1016/S0005-2728(99)00024-9 10320667

[phy270331-bib-0011] Dias, C. A. , Souza‐Costa, D. C. , Zerbini, T. , da Rocha, J. B. , Gerlach, R. F. , & Tanus‐Santos, J. E. (2005). The effect of sildenafil on pulmonary embolism‐induced oxidative stress and pulmonary hypertension. Anesthesia and Analgesia, 101(1), 115–120. 10.1213/01.ANE.0000153499.10558.F3 15976216

[phy270331-bib-0012] Dundon, B. K. , Torpey, K. , Nelson, A. J. , Wong, D. T. , Duncan, R. F. , Meredith, I. T. , Faull, R. J. , Worthley, S. G. , & Worthley, M. I. (2014). The deleterious effects of arteriovenous fistula‐creation on the cardiovascular system: A longitudinal magnetic resonance imaging study. International Journal of Nephrology and Renovascular Disease, 7, 337–345. 10.2147/IJNRD.S66390 25258554 PMC4172192

[phy270331-bib-0013] Gold, J. A. , & Hoffman, K. (2006). Fistula first: The National Vascular Access Improvement Initiative. Wisconsin Medical Journal, 105, 71–73. 16749331

[phy270331-bib-0014] Gorecka, J. , Fereydooni, A. , Gonzalez, L. , Lee, S. R. , Liu, S. , Ono, S. , Xu, J. , Liu, J. , Taniguchi, R. , Matsubara, Y. , Gao, X. , Gao, M. , Langford, J. , Yatsula, B. , & Dardik, A. (2019). Molecular targets for improving arteriovenous fistula maturation and patency. Vascular Investigation and Therapy, 2(2), 33. 10.4103/VIT.VIT_9_19 31608322 PMC6788624

[phy270331-bib-0015] He, Y. , Shiu, Y. T. , Imrey, P. B. , Radeva, M. K. , Beck, G. J. , Gassman, J. J. , Northrup, H. M. , Roy‐Chaudhury, P. , Berceli, S. A. , Cheung, A. K. , & for the Hemodialysis Fistula Maturation (HFM) Study Group* . (2023). Association of Shear Stress with subsequent lumen remodeling in hemodialysis arteriovenous fistulas. Clinical Journal of the American Society of Nephrology, 18, 72–83. 36446600 10.2215/CJN.04630422 PMC10101625

[phy270331-bib-0016] Hirschberg, K. , Radovits, T. , Loganathan, S. , Entz, L. , Beller, C. J. , Gross, M.–. L. , Sandner, P. , Karck, M. , & Szabó, G. (2009). Selective phosphodiesterase‐5 inhibition reduces neointimal hyperplasia in rat carotid arteries after surgical endarterectomy. The Journal of Thoracic and Cardiovascular Surgery, 137, 1508–1514. 19464472 10.1016/j.jtcvs.2008.10.016

[phy270331-bib-0017] Kniotek, M. , Roszczyk, A. , Zych, M. , Wrzosek, M. , Szafarowska, M. , Zagożdżon, R. , & Jerzak, M. (2021). Sildenafil citrate downregulates pde5a mrna expression in women with recurrent pregnancy loss without altering angiogenic factors—A preliminary study. Journal of Clinical Medicine, 10, 5086. 34768607 10.3390/jcm10215086 PMC8584603

[phy270331-bib-0018] Krishnamoorthy, M. K. , Banerjee, R. K. , Wang, Y. , Zhang, J. , Roy, A. S. , Khoury, S. F. , Arend, L. J. , Rudich, S. , & Roy‐Chaudhury, P. (2008). Hemodynamic wall shear stress profiles influence the magnitude and pattern of stenosis in a pig AV fistula. Kidney International, 74, 1410–1419. 18818686 10.1038/ki.2008.379

[phy270331-bib-0019] Langer, S. , Kokozidou, M. , Heiss, C. , Kranz, J. , Kessler, T. , Paulus, N. , Krüger, T. , Jacobs, M. J. , Lente, C. , & Koeppel, T. A. (2010). Chronic kidney disease aggravates arteriovenous fistula damage in rats. Kidney International, 78, 1312–1321. 20881937 10.1038/ki.2010.353

[phy270331-bib-0020] Lee, T. (2013). Novel paradigms for dialysis vascular access: Downstream vascular biology‐is there a final common pathway? Clinical Journal of the American Society of Nephrology, 8, 2194–2201. 23990166 10.2215/CJN.03490413 PMC3848398

[phy270331-bib-0021] Lee, T. , Somarathna, M. , Hura, A. , Wang, Y. , Campos, B. , Arend, L. , Munda, R. , & Roy‐Chaudhury, P. (2014). Natural history of venous morphologic changes in dialysis access stenosis. The Journal of Vascular Access, 15, 298–305. 24500849 10.5301/jva.5000212

[phy270331-bib-0022] Lin, C. S. (2004). Tissue expression, distribution, and regulation of PDE5. International Journal of Impotence Research, 16, S8–S10. 15224128 10.1038/sj.ijir.3901207

[phy270331-bib-0023] Lok, C. E. , Huber, T. S. , Lee, T. , Shenoy, S. , Yevzlin, A. S. , Abreo, K. , Allon, M. , Asif, A. , Astor, B. C. , Glickman, M. H. , Graham, J. , Moist, L. M. , Rajan, D. K. , Roberts, C. , Vachharajani, T. J. , Valentini, R. P. , National Kidney Foundation , & Vascular Access Guideline Work Group . (2020). KDOQI clinical practice guideline for vascular access: 2019 update. American Journal of Kidney Diseases, 75(4 Suppl 2), S1–S164. 10.1053/j.ajkd.2019.12.001 32778223

[phy270331-bib-0024] Lugnier, C. (2006). Cyclic nucleotide phosphodiesterase (PDE) superfamily: A new target for the development of specific therapeutic agents. Pharmacology and Therapeutics, 109(3), 366–398. 10.1016/j.pharmthera.2005.07.003 16102838

[phy270331-bib-0025] Martinez, L. , Duque, J. C. , Tabbara, M. , Paez, A. , Selman, G. , Hernandez, D. R. , Sundberg, C. A. , Tey, J. C. S. , Shiu, Y. T. , Cheung, A. K. , Allon, M. , Velazquez, O. C. , Salman, L. H. , & Vazquez‐Padron, R. I. (2018). Fibrotic venous remodeling and nonmaturation of arteriovenous fistulas. Journal of the American Society of Nephrology, 29, 1030–1040. 29295872 10.1681/ASN.2017050559 PMC5827597

[phy270331-bib-0026] Massaro, M. , Scoditti, E. , Pellegrino, M. A. , Calabriso, N. , Carluccio, M. A. , Storelli, C. , & de Caterina, R. (2014). P739Phosphodiesterase 5A expression is up‐regulated in vascular endothelium under pro‐inflammatory conditions: A newly disclosed anti‐inflammatory activity by the omega‐3 fatty acid docosahexaenoic acid. Cardiovascular Research, 103, S135.

[phy270331-bib-0027] Montfort, W. R. , Wales, J. A. , & Weichsel, A. (2017). Structure and activation of soluble Guanylyl cyclase, the nitric oxide sensor. Antioxidants & Redox Signaling, 26(3), 107–121. 10.1089/ars.2016.6693 26979942 PMC5240008

[phy270331-bib-0028] Mortamais, J. , Papillard, M. , Girouin, N. , Boutier, R. , Cougnaud, L. , Martin, X. , Badet, L. , Juillard, L. , & Rouvière, O. (2013). Endovascular treatment of juxta‐anastomotic venous stenoses of forearm radiocephalic fistulas: Long‐term results and prognostic factors. Journal of Vascular and Interventional Radiology, 24, 558–564. 23384833 10.1016/j.jvir.2012.12.004

[phy270331-bib-0029] Northrup, H. , He, Y. , le, H. , Berceli, S. A. , Cheung, A. K. , & Shiu, Y. T. (2022). Differential hemodynamics between arteriovenous fistulas with or without intervention before successful use. Frontiers in Cardiovascular Medicine, 9, 1001267. 36407418 10.3389/fcvm.2022.1001267 PMC9669082

[phy270331-bib-0030] Northrup, H. , Somarathna, M. , Corless, S. , Falzon, I. , Totenhagen, J. , Lee, T. , & Shiu, Y. T. (2021). Analysis of geometric and hemodynamic profiles in rat arteriovenous fistula following PDE5A inhibition. Frontiers in Bioengineering and Biotechnology, 9, 779043. 34926425 10.3389/fbioe.2021.779043 PMC8675087

[phy270331-bib-0031] Omori, K. , & Kotera, J. (2007). Overview of PDEs and their regulation. Circulation Research, 100, 309–327. 10.1161/01.RES.0000256354.95791.f1 17307970

[phy270331-bib-0032] Ori, Y. , Korzets, A. , Katz, M. , Erman, A. , Weinstein, T. , Malachi, T. , & Gafter, U. (2002). The contribution of an arteriovenous access for hemodialysis to left ventricular hypertrophy. American Journal of Kidney Diseases, 40, 745–752. 12324909 10.1053/ajkd.2002.35685

[phy270331-bib-0033] Pike, D. , Shiu, Y. T. , Cho, Y. F. , le, H. , Somarathna, M. , Isayeva, T. , Guo, L. , Symons, J. D. , Kevil, C. G. , Totenhagen, J. , & Lee, T. (2019). The effect of endothelial nitric oxide synthase on the hemodynamics and wall mechanics in murine arteriovenous fistulas. Scientific Reports, 9, 4299. 10.1038/s41598-019-40683-7 30862797 PMC6414641

[phy270331-bib-0034] Pike, D. , Shiu, Y. T. , Somarathna, M. , Guo, L. , Isayeva, T. , Totenhagen, J. , & Lee, T. (2017). High resolution hemodynamic profiling of murine arteriovenous fistula using magnetic resonance imaging and computational fluid dynamics. Theoretical Biology & Medical Modelling, 14, 5. 10.1186/s12976-017-0053-x 28320412 PMC5360029

[phy270331-bib-0035] Rajabi‐Jagahrgh, E. , Krishnamoorthy, M. K. , Wang, Y. , Choe, A. , Roy‐Chaudhury, P. , & Banerjee, R. K. (2013). Influence of temporal variation in wall shear stress on intima‐media thickening in arteriovenous fistulae. Seminars in Dialysis, 26, 511–519. 23278290 10.1111/sdi.12045

[phy270331-bib-0036] Rao, N. N. , Stokes, M. B. , Rajwani, A. , Ullah, S. , Williams, K. , King, D. , Macaulay, E. , Russell, C. H. , Olakkengil, S. , Carroll, R. P. , Faull, R. J. , Teo, K. S. L. , McDonald, S. P. , Worthley, M. I. , & Coates, P. T. (2019). Effects of arteriovenous fistula ligation on cardiac structure and function in kidney transplant recipients. Circulation, 139, 2809–2818. 31045455 10.1161/CIRCULATIONAHA.118.038505

[phy270331-bib-0037] Rothuizen, T. C. , Wong, C. , Quax, P. H. , van Zonneveld, A. J. , Rabelink, T. J. , & Rotmans, J. I. (2013). Arteriovenous access failure: More than just intimal hyperplasia? Nephrology, Dialysis, Transplantation, 28(5), 1085–1092. 10.1093/ndt/gft068 23543595

[phy270331-bib-0038] Roy‐Chaudhury, P. , Arend, L. , Zhang, J. , Krishnamoorthy, M. , Wang, Y. , Banerjee, R. , Samaha, A. , & Munda, R. (2007). Neointimal hyperplasia in early arteriovenous fistula failure. American Journal of Kidney Diseases, 50, 782–790. 17954291 10.1053/j.ajkd.2007.07.019

[phy270331-bib-0039] Sands, J. J. (2000). Increasing AV fistulas: Revisiting a time‐tested solution. Seminars in Dialysis, 13, 351–353. 11130254 10.1046/j.1525-139x.2000.00098.x

[phy270331-bib-0040] Sasser, J. M. , & Baylis, C. (2010). Effects of sildenafil on maternal hemodynamics and fetal growth in normal rat pregnancy. American Journal of Physiology. Regulatory, Integrative and Comparative Physiology, 298, R433–R438. 19955496 10.1152/ajpregu.00198.2009 PMC2828177

[phy270331-bib-0041] Schäfer, M. , Barker, A. J. , Kheyfets, V. , Stenmark, K. R. , Crapo, J. , Yeager, M. E. , Truong, U. , Buckner, J. K. , Fenster, B. E. , & Hunter, K. S. (2017). Helicity and vorticity of pulmonary arterial flow in patients with pulmonary hypertension: Quantitative analysis of flow formations. Journal of the American Heart Association, 6, e007010. 29263034 10.1161/JAHA.117.007010 PMC5779020

[phy270331-bib-0042] Shiu, Y. T. , Rotmans, J. I. , Geelhoed, W. J. , Pike, D. B. , & Lee, T. (2019). Arteriovenous conduits for hemodialysis: How to better modulate the pathophysiological vascular response to optimize vascular access durability. American Journal of Physiology. Renal Physiology, 316, F794–F806. 10.1152/ajprenal.00440.2018 30785348 PMC6580244

[phy270331-bib-0043] Sivanesan, S. , How, T. V. , & Bakran, A. (1998). Characterizing flow distributions in AV fistulae for haemodialysis access. Nephrology, Dialysis, Transplantation, 13(12), 3108–3110. 10.1093/ndt/13.12.3108 9870474

[phy270331-bib-0044] Somarathna, M. , Hwang, P. T. , Millican, R. C. , Alexander, G. C. , Isayeva‐Waldrop, T. , Sherwood, J. A. , Brott, B. C. , Falzon, I. , Northrup, H. , Shiu, Y. T. , Stubben, C. J. , Totenhagen, J. , Jun, H. W. , & Lee, T. (2022). Nitric oxide releasing nanomatrix gel treatment inhibits venous intimal hyperplasia and improves vascular remodeling in a rodent arteriovenous fistula. Biomaterials, 280, 121254. 10.1016/j.biomaterials.2021.121254 34836683 PMC8724452

[phy270331-bib-0045] United States Renal Data System . (2022). USRDS Annual Data Report: Epidemiology of kidney disease in the United States. *Natl. Institutes Heal. Natl. Inst. Diabetes Dig. Kidney Dis* .

[phy270331-bib-0046] Vazquez‐Padron, R. I. , Duque, J. C. , Tabbara, M. , Salman, L. H. , & Martinez, L. (2021). Intimal hyperplasia and arteriovenous fistula failure: Looking beyond size differences. Kidney360, 2, 1360–1372. 34765989 10.34067/KID.0002022021 PMC8579754

[phy270331-bib-0047] Veres, G. , Hagenhoff, M. , Schmidt, H. , Radovits, T. , Loganathan, S. , Bai, Y. , Korkmaz‐Icöz, S. , Brlecic, P. , Sayour, A. A. , Karck, M. , & Szabó, G. (2018). Targeting Phosphodiesterase‐5 by Vardenafil improves vascular graft function. European Journal of Vascular and Endovascular Surgery, 56, 256–263. 29724533 10.1016/j.ejvs.2018.03.025

[phy270331-bib-0048] Yang, H. M. , Jin, S. , Jang, H. , Kim, J. Y. , Lee, J. E. , Kim, J. , & Kim, H. S. (2019). Sildenafil reduces Neointimal hyperplasia after angioplasty and inhibits platelet aggregation via activation of cGMP‐dependent protein kinase. Scientific Reports, 9, 7769. 31123275 10.1038/s41598-019-44190-7 PMC6533301

