# Supplementary material for: Vascular remodeling in arteriovenous fistula treated with PDE5A inhibitors
Source: Physiol Rep. 2025 Apr 29;13(9):e70331. doi: 10.14814/phy2.70331 (PMC12040441; doi:10.14814/phy2.70331)
Supplement: Supplementary file 1 — Figure S1. [file PHY2-13-e70331-s001.pdf]

**SUPPLEMENTARY FIGURES**

## Vascular remodeling in arteriovenous fistula treated with PDE5A inhibitors

*Maheshika Somarathna[1], *Hannah Northrup[2], Kevin Ingle[1], Tatyana Isayeva-Waldrop[1], Nguyen Thuy Nhu Nguyen[1], Bailey Lose[1], Yan-Ting Shiu[2,3,4], and Timmy Lee[1,5]

Affiliations: Department of Medicine and Division of Nephrology, University of Alabama at Birmingham, AL[1]; Department of Internal Medicine and Division of Nephrology and Hypertension, University of Utah, Salt Lake, UT[2]; Veterans Affairs Medical Center, Salt Lake City, UT[3]; Nora Eccles Harrison Cardiovascular Research and Training Institute, University of Utah, Salt Lake City, UT[4]; Veterans Affairs Medical Center, Birmingham, AL[5]

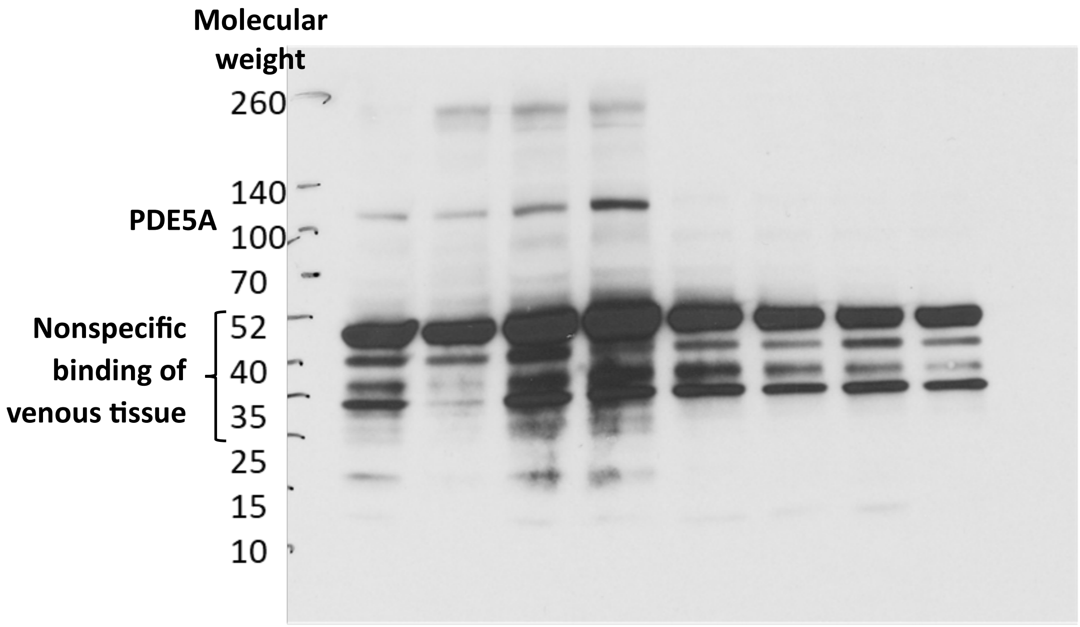


**Supplemental Figure 1: Full length western blot gel corresponding to Figure 1B PDE5A.**

**Molecular weight**

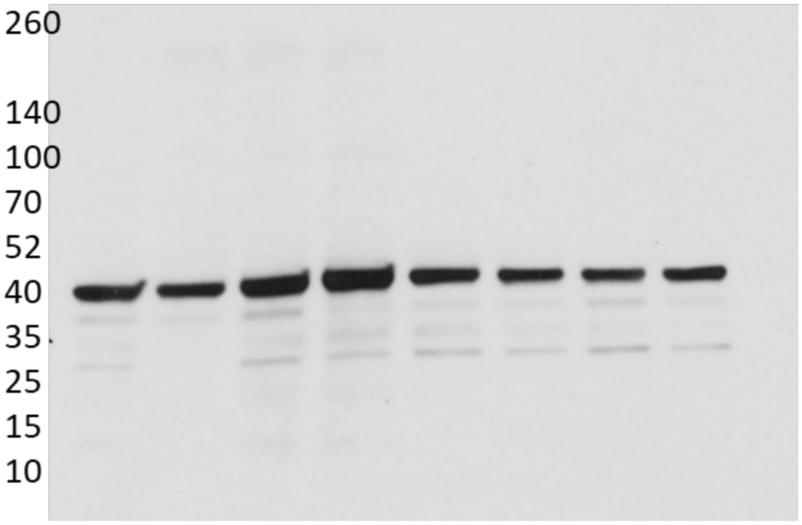


**Supplemental Figure 2: Full length western blot gel corresponding to Figure 1B GAPDH; re-probe of Supplemental Figure 1.**
